# Potential application of novel technology developed for instant decontamination of personal protective equipment before the doffing step

**DOI:** 10.1371/journal.pone.0250854

**Published:** 2021-06-04

**Authors:** Luís Alberto Brêda Mascarenhas, Bruna Aparecida Souza Machado, Leticia de Alencar Pereira Rodrigues, Katharine Valéria Saraiva Hodel, Alex Álisson Bandeira Santos, Paulo Roberto Freitas Neves, Leone Peter Correia da Silva Andrade, Milena Botelho Soares, Jailson Bittencourt de Andrade, Roberto Badaró

**Affiliations:** 1 SENAI CIMATEC, SENAI Institute of Innovation (ISI) in Health Advanced Systems (CIMATEC ISI SAS), University Center SENAI/CIMATEC, Salvador, Bahia, Brazil; 2 SENAI CIMATEC, National Service of Industrial Learning–SENAI, Computational Modeling and Industrial Technology, University Center SENAI/CIMATEC, Salvador, Bahia, Brazil; 3 Gonçalo Moniz Institute, Oswaldo Cruz Foundation (IGM-FIOCRUZ/BA), Salvador, Bahia, Brazil; VIT University, INDIA

## Abstract

The use of personal protective equipment (PPE) has been considered the most effective way to avoid the contamination of healthcare workers by different microorganisms, including SARS-CoV-2. A spray disinfection technology (chamber) was developed, and its efficacy in instant decontamination of previously contaminated surfaces was evaluated in two exposure times. Seven test microorganisms were prepared and inoculated on the surface of seven types of PPE (respirator mask, face shield, shoe, glove, cap, safety glasses and lab coat). The tests were performed on previously contaminated PPE using a manikin with a motion device for exposure to the chamber with biocidal agent (sodium hypochlorite) for 10 and 30s. In 96.93% of the experimental conditions analyzed, the percentage reduction was >99% (the number of viable cells found on the surface ranged from 4.3x10^6^ to <10 CFU/mL). The samples of *E*. *faecalis* collected from the glove showed the lowest percentages reduction, with 86.000 and 86.500% for exposure times of 10 and 30 s, respectively. The log_10_ reduction values varied between 0.85 log_10_ (*E*. *faecalis* at 30 s in glove surface) and 9.69 log_10_ (*E*. *coli* at 10 and 30 s in lab coat surface). In general, *E*. *coli*, *S*. *aureus*, *C*. *freundii*, *P*. *mirabilis*, *C*. *albicans* and *C*. *parapsilosis* showed susceptibility to the biocidal agent under the tested conditions, with >99% reduction after 10 and 30s, while *E*. *faecalis* and *P*. *aeruginosa* showed a lower susceptibility. The 30s exposure time was more effective for the inactivation of the tested microorganisms. The results show that the spray disinfection technology has the potential for instant decontamination of PPE, which can contribute to an additional barrier for infection control of healthcare workers in the hospital environment.

## Introduction

Contaminated surfaces are a potential source for the spread of many bacterial and fungal pathogens [1]. These microorganisms can be considered important vectors for the dissemination of diseases and, consequently, the increase in mortality and morbidity rates, causing overload of the health system worldwide [2]. There is currently growing concern that the environment may be an underestimated source for the spread of emerging viruses, including of the influenza virus [3], Ebola virus [4], and coronaviruses, especially the severe acute respiratory syndrome named SARS-CoV-2 [5]. SARS-CoV-2 is the causative agent of novel coronavirus 2019 disease (COVID-19) which was isolated and identified for the first time in humans in the city of Wuhan, Hubei Province, China [6]. Based on evidence of an increasing incidence of infections [7] and the possibility of transmission by asymptomatic carriers [8], it was demonstrated that SARS-CoV-2 can be effectively transmitted between humans through droplets (aerosols) or direct contact with contaminated surfaces, which facilitated its rapid spread worldwide [9, 10].

Healthcare workers (HCWs) are one of the most vulnerable populations to microbial contamination, mainly because they work in close physical contact with patients [11]. This vulnerability was demonstrated at times of emergency in health systems, such as during the outbreak caused by SARS-CoV [12], Ebola virus [13] and currently with SARS-CoV-2 [14], where a high rate of infection among HCWs has been reported. The high prevalence of COVID-19 among HCWs is mainly associated with the execution of the procedures involved in airway management for oxygen supplementation of many patients with severe COVID-19 pneumonia presenting with pronounced arterial hypoxemia (major generators of aerosol) [12, 15], which increases the viral load in which these professionals are in contact [16, 17]. The risk of viral transmission to HCWs has been a concern since the beginning of the outbreak in China, where more than 3,300 HCWs were infected, with a mortality rate of 1.1% [18]. In Europe, approximately 20% of HCWs were infected by SARS-CoV-2 in Italy and 26% in Spain, the two epicenters of the disease in the European continent between March and April [19, 20]. In Brazil, currently considered the epicenter of the disease in Latin America [21], data from the Ministry of Health indicate that at least 257,156 HCWs were infected by SARS-CoV-2 by August of this year [22].

The use of personal protective equipment (PPE) by HCWs has been considered the most effective way to avoid contamination by different microorganisms of high epidemiological concern [23–25], including SARS-CoV-2 [26], as they have the ability to act as a barrier to pathogens [27]. Studies have shown that the use of PPE and actions to decontaminate their surfaces are crucial to reduce the infection rate among HCWs in direct contact with patients diagnosed with COVID-19 and other contagious diseases [23, 28]. The step of PPE removal (doffing) by HCWs can be thus considered critical since there may be contact between the contaminated surface of the PPE and the HCWs, leading to an increased chance of self-contamination through the mucous membranes of the nose, eyes or mouth [29]. Therefore, this step should be performed following well-established biosafety protocols [30], It has been demonstrated that doffing PPE is among others an important risk factor associated with HCWs contamination with SARS-CoV-2 [31].

Several devices with different technologies have been developed for the inactivation or reduction of bioburden on surfaces and environments [1], and the use of such devices has gained popularity for presenting a response to the global demand created for the control of possible environmental surfaces contamination [32]. Examples include devices with ultraviolet-C (UV-C) or xenon UV light for disinfection of hospital environments [33, 34], portable equipment with a disinfectant spraying system [35] or hospital air disinfection systems [36] and disinfection chambers [37]. Faced with increased production and demand, health regulatory agencies recommend that studies be presented to prove the performance of these devices in the face of decontamination effectiveness [38, 39]. Although there are few studies demonstrating the efficacy of disinfection chambers [40], their use becomes interesting for the decontamination of surfaces, including personal protective closes and equipment (PPE), since it is not mandatory that the contaminated material undergoes to manual cleaning mainly at the moment before the doffing step for previous elimination of the microorganisms [37]. Thus, disinfection chambers can be a practical alternative for bioburden control in environments with a high rate of pathogenic microorganisms, such as hospitals. In addition, the disinfection chambers can help mitigate the possibility of an accident self-contamination of the HCWs during the processing of hand manipulation of the disposable PPEs.

Different disinfecting agents have been studied and suggested to act in these devices as biocidal agents against different pathogens of hospital importance (such SARS-CoV-2, multidrug-resistant bacteria and fungi). Examples include physical agents such as UV light [41] and chemical agents such as alcoholic solutions [5, 42], quaternary ammonium compounds [43, 44], ozone gas [45] and sodium hypochlorite [46]. Sodium hypochlorite is one of the most well-known and used biocidal agents worldwide due to its broad-spectrum microbicidal properties [47]. Compared with other chlorine-containing biocides, the use of sodium hypochlorite is characterized by a relatively lower toxicity when in contact with mucous membranes, the equipment required for its synthesis are simpler, its handling is safer, and its operation and preparation costs are lower, which makes its use feasible in hospitals [48]. In addition, the use of sodium hypochlorite is recommended by the World Health Organization (WHO) for the disinfection of environmental surfaces related to health care in the context of COVID-19, in concentrations between 0.1 and 0.5% (1000 and 5000 ppm, respectively) [49].

Despite being a promising alternative, especially considering the current situation, there are still few reports in the literature on the efficacy of disinfection chambers and/or devices using sodium hypochlorite as a biocidal agent for reducing or inactivating the burden of different pathogens on contaminated surfaces, more specifically for PPE, and on its potential use in emergency and public health situations. [38, 50, 51]. The use of disinfection chambers in controlled environments, such as hospitals and health units, could help to reduce the risk of self-contamination by health workers during the doffing step, since the instantaneously dispersed solution could significantly reduce the pathogens present on surfaces and contribute to greater safety of these HCWs self-contamination. Nevertheless, this approach has not yet been reported by any earlier study.

Through the use of the disinfection chamber it is possible to decontaminate the surfaces of all PPEs used in clinical practice at the same time, making the doffing step safer for the HCWs. This can be considered an advantage over the PPE disinfection technologies mentioned in the literature [52–54], since the proposed decontamination processes do not reduce the risk of self-contamination. Indeed, the objective of this study was to develop a disinfection chamber for instantaneous dispersion of a biocidal solution (0.25% sodium hypochlorite) and to determine its efficacy on previously contaminated surfaces at different exposure times, aiming at its possible application as an additional barrier against pathogens, such as SARS-CoV-2, to protect HCWs during the withdraw procedure prior PPE disposal.

## Materials and methods

**Fig 1:**
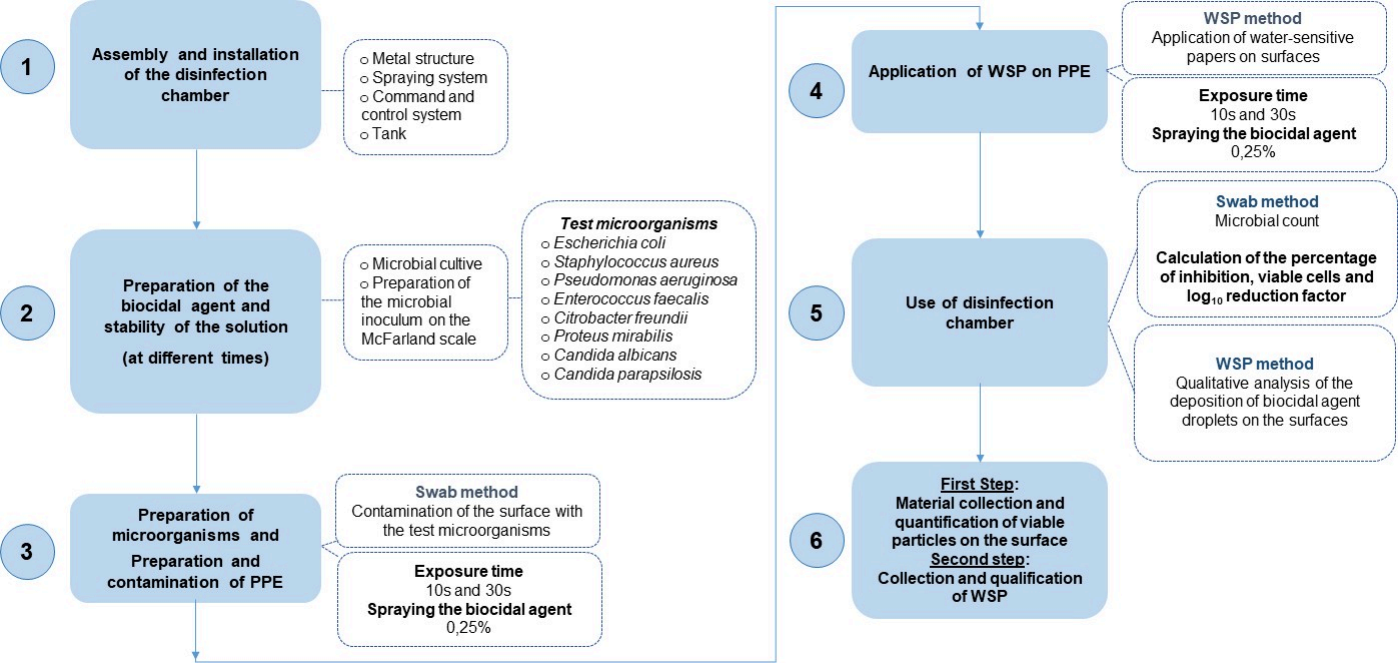
Schematic illustrating the method used in this study to evaluate the spray disinfection technology for instant decontamination of personal protective equipment.

Fig 1 shows the general scheme of the method applied in this study to evaluate the efficacy of the chamber for instant disinfection of the surfaces of seven PPE previously contaminated with different microorganisms and subjected to different exposure times to the biocidal agent (0.25% sodium hypochlorite). To evaluate the efficacy of the disinfection process, tests were performed in two distinct steps with quantitative and qualitative analyses using a manikin that moved through a linear and rotary motion system to simulate the passage of an individual in a hospital environment (before the doffing step), an environment well known for presenting a high burden of infectious agents [55].

### Development and installation of the disinfection chamber

The chamber consisted of a modular framework constructed of aluminum and carbon steel, with dimensions of 240 x 150 x 250 cm (height x depth x width), with an open entrance and exit. The design of the disinfection chamber allows the framework to be easily transported, installed and uninstalled. The nebulization system for the chamber comprised six nebulizer nozzles (Senninger, USA) installed on the inner side and top of the chamber to promote a better homogeneity in the spraying of the biocide agent. In addition, a water filter, a submerged pump and a 1-m^3^ storage tank (1000 L capacity) with lid, with flow rate of 10 L/h were used to complete the system. The command and control system was installed by means of an electrical panel with voltage of 220 V, was responsible for the activation and operation of the entire system and contained a sensor for the activation of the nebulizers.

### Biocidal agent: Preparation and stability analysis

The biocidal agent (bleach) was prepared at a concentration of 0.25% [49, 56] by diluting an initial solution with 2.38% active chlorine [57]. A total volume of 1,000 L was prepared directly in the storage tank of the chamber for the experiments. All experiments to evaluate the disinfection potential of PPE were performed during the first three days after preparation of the biocidal agent (bleach). The stability of the biocidal agent (0.25% sodium hypochlorite) was evaluated on days 0, 3, 6, 9, 13 and 20 by determining the percentage of active chlorine present in the solution and through pH analysis. The use of the biocide agent in the concentration of 0.25% was based on the WHO recommendation for the disinfection of environmental surfaces, which ranges from 0.1 to 0.5% [49]. Thus, an intermediate concentration was chosen. The amount of active chlorine was evaluated by iodometric titration [58], and the pH was determined through the hydrogen ionic activity using a standard electrode (pH meter, Mettler-Toledo). The analyses were performed in triplicates.

### Experimental standard strains

The standard reference strains used in this study were *Escherichia coli* (ATCC 8739), *Staphylococcus aureus* (ATCC 6538), *Pseudomonas aeruginosa* (ATCC 27853), *Enterococcus faecalis* (ATCC 29212), *Citrobacter freundii* (ATCC 43864), *Proteus mirabilis* (ATCC 29906), *Candida albicans* (ATCC 18804) and *Candida parapsilosis* (ATCC 22019), which were obtained from Microbiologics (St. Cloud, Minnesota) or from the Culture Collection of the Institute of Health Sciences, Federal University of Bahia (Universidade Federal da Bahia–UFBA), located in Salvador, Brazil. The selection of test strains was based on studies of microorganisms commonly causing nosocomial infections, as well as on the recommendations of regulatory agencies for evaluating the efficacy of chemical disinfectants [57, 59–65]. The suspensions of the test microorganisms were prepared by transferring cells from the pure culture to plates containing 15–20 mL of plate count agar: agar (9 g/L); dextrose (1 g/L); tryptone (5.0 g/L) and yeast extract (2.5 g/L). To evaluate the disinfection profile in the chamber against the test microorganisms, the inocula were prepared by suspending 1–5 colonies in 5 mL of 0.85% saline solution and the turbidity was adjusted to McFarland No. 0.5 tube [66].

### Preparation of study surfaces (PPE)

The PPE items used to evaluate the effectiveness of the disinfection chamber were selected according to the recommendations for prevention and control of the spread of SARS-CoV-2 and other infectious agents transmitted mainly by aerosols in health services (Table 1) [24, 67, 68]. To ensure the sterility of the surface of the selected items before contamination with the standard strains, the items were exposed to UV light for 40 minutes using a laminar flow (model LA2000T, LOGEN) after being sanitized with 70% ethanol [69]. Surface samples from each item were collected using sterile swabs, and their contents were seeded in nutrient agar (37°C for 24 hours) to confirm sterility.

**Table 1:** Items used to evaluate the efficacy of instantaneous spraying of biocidal agent (0.25% sodium hypochlorite) in a disinfection chamber against the test microorganisms.

Selected item	Brand	Composition	Surface type
Respirator face mask	Air Safety	Polypropylene	Porous
Professional shoe	Soft Works	Ethylene vinyl acetate	Nonporous
Procedure glove	Supermax	Nitrile (nitrile)	Porous
Disposable Cap	Descarpack	Polypropylene	Porous
Face shield	CIMATEC	Polycarbonate	Nonporous
Safety glasses	Carbography	Polycarbonate	Nonporous
Disposable lab coat (apron)	Jarc Smart Products	Polypropylene and Polyethylene	Porous

### Assay for evaluation and distribution of the biocidal agent for spray disinfection during exposure in the chamber

The assays for evaluating the disinfection potential of the biocidal agent in the chamber developed in this study were based on the method used to monitor viable particles on surfaces [70, 71] and qualitative analysis of the biocidal agent distribution on the surfaces [72]. The disinfection process was performed by spraying the biocidal agent (0.25% sodium hypochlorite) in the chamber using a suitably dressed manikin with a motion system that allowed the manikin to pass through the chamber automatically and to perform a 360° turn, for 10 and 30 s of exposure. The manikin was chosen so that there would be a simulation closer to what would be the use of the disinfection chamber by healthcare workers in nosocomial environment. In the first step, the surfaces were contaminated using a sterile swab immersed in the test tube containing the test microorganism.

**Fig 2:**
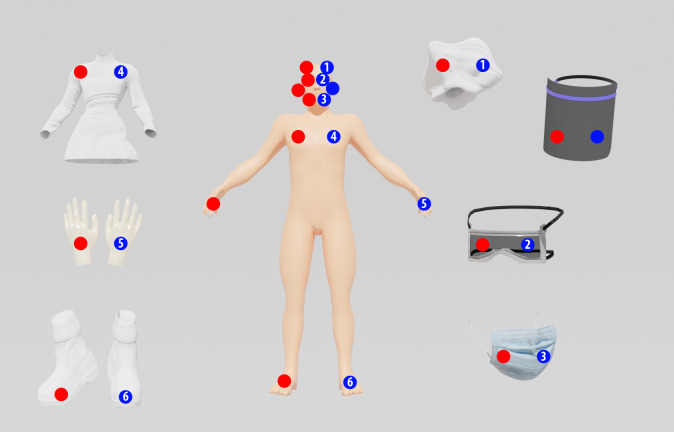
Demarcated areas for contamination by the test microorganisms for evaluation of the potential of instantaneous disinfection by the spray biocidal agent (0.25% sodium hypochlorite). The red circles represent the inoculation area (30 cm^2^) used as control (right side), the blue circles represent the inoculation area (30 cm^2^) used as test (left side), and the numbers in the blue circles represent the positions of the water-sensitive papers for each experiment: (1) cap; (2) safety glasses; (3) respirator face mask; (4) lab coat; (5) glove; and (6) shoe. For the control, the surface of the items was swabbed for microorganism collection immediately after surface contamination, while for the tests, the surfaces were swabbed after predetermined exposure times to the biocidal agent.

Previously demarcated areas of 30 cm^2^ [70] were used for contamination, with the right side used for the control (without exposure to the biocidal agent by spraying, in other words, these surfaces were sampled before the manikin (and PPE) was decontaminated) and the left side used for each test microorganism (Fig 2). The surface of the contaminated item was sampled with a swab immersed in 10 mL of buffered peptone water (Swab-Samplers - 3M, USA), and its content was used to analyze the number of viable particles on the surface for the control and after exposure to the disinfection chamber. For the control test, swabbing for microorganism collection was performed immediately after contamination; for the exposure tests to the biocidal agent, after 10 and 30 s. The control surfaces of each PPE analyzed were associated with both exposure experiments (10 and 30 s exposure). The antimicrobial action of sodium hypochlorite was neutralized by sodium thiosulphate [73, 74]. Sterile PPE items were used for each experiment, with a new item used for each microorganism or exposure time. In the second step, water-sensitive papers (WSP) (76x26 mm and 19.76 cm^2^ area; Syngenta) were labeled from 1 to 6 and applied to the left side of the manikin (Fig 2) for exposure to the biocidal agent using the same exposure times and contamination sites of the first experiment. After each experiment, the papers were immediately stored in a desiccator with silica gel, and then images were recorded for qualitative analysis (observation of the paper color profile) of the deposition of biocidal agent on the surfaces of the PPE items.

### Monitoring of viable particles on the surface

Viable microorganisms in the swabbed samples were determined using a nutrient agar culture method specific for each type of microorganism, which were quantified from their growth in the plate [75]. The tests were performed immediately after the swabbed samples were collected. The samples were vigorously shaken to extract the microorganisms from the swab and release them into the saline solution so that they could be serially diluted (10^−1^ to 10^−8^). The dilutions were inoculated into the specific culture media and incubated according to the type of method. For *E*. *coli*, *P*. *mirabilis* and *C*. *freundii*, the VRBA count method was used; for *P*. *aeruginosa*, *S*. *aureus*, *C*. *albicans* and *C*. *parapsilosis*, the count of the total number of mesophilic microorganisms; and *E*. *faecalis* were counted by the EPA (US Environmental Protection Agency) method [70, 76, 77]. After quantification of the colonies under an optical microscope (Nikon Instruments), the results were expressed as log_10_ CFU/mL and CFU/cm^2^. The number of CFUs was determined after incubation, and the number of CFUs per milliliter was calculated. The logarithmic scale (log_10_) reduction factor was calculated using the formula RF = log_10_ (A)—log_10_ (B) (where A is the number of colonies recovered from the unexposed (control) surfaces and B is the number of colonies recovered from the exposed (test) surfaces) [56, 78]. The decimal percentage reduction in CFU/mL was calculated using the formula %R = [(A—B)/A] * 100 [79].

### Statistical analysis

Statistical analysis was performed using GraphPad Prism 8 (San Diego, CA, USA), where analysis of variance and Student’s t-test were used to compare the means of the two groups (10 and 30 s), according to each test condition (microorganism x PPE item), with significance level of p <0.05. Principal component analysis (PCA) was performed using PAST version 3.26 (Oslo, Norway) with the means of the logarithmic reductions of each test condition to obtain the correlation between the analyzed variables (PPE–cap, safety glasses, respirator face mask, lab coat, glove, shoe and face shield–or surface type).

## Results

**Fig 3:**
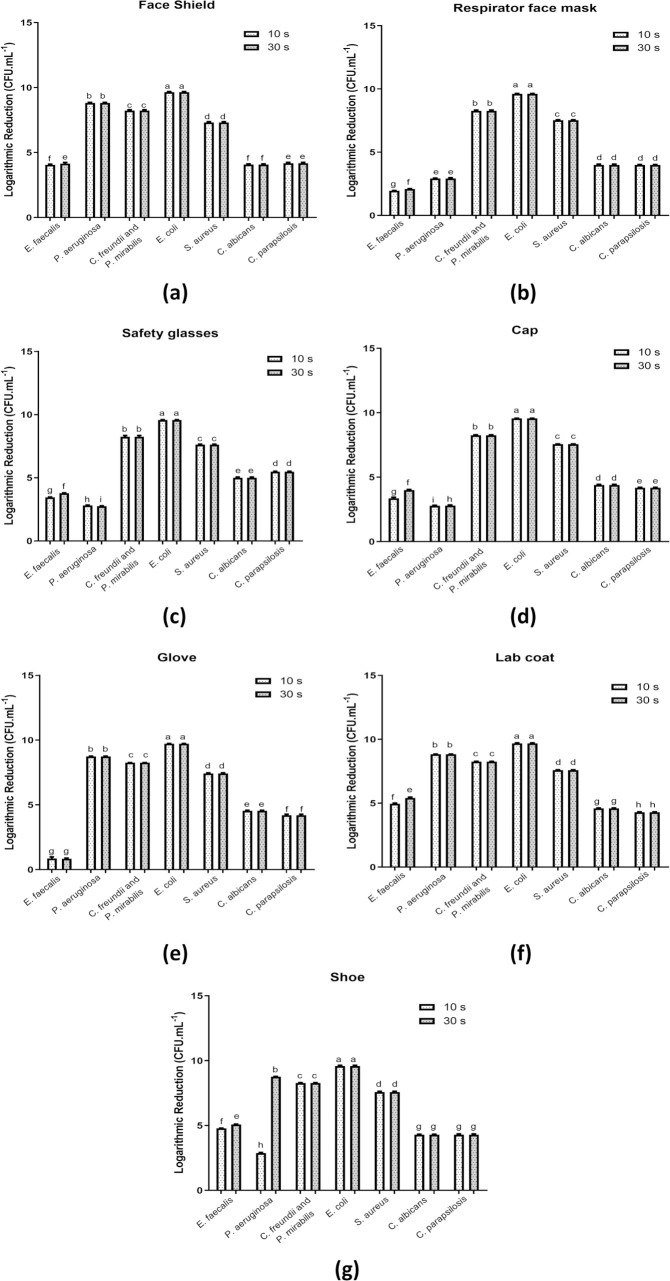
Logarithmic reduction of the analyzed test microorganisms after exposure to the biocidal agent for 10 and 30 s according to the PPE item: (a) face shield; (b) respirator face mask; (c) safety glasses; (d) cap; (e) glove (f) lab coat and (g) shoe. Bars followed by the same letters are not significantly different at p <0.05 according to Student’s t test with 95% confidence.

Table 2 shows the results for the number of viable cells (CFU/mL and CFU/cm^2^), the logarithmic reduction factor (log_10_) of each assay compared to the respective control (without exposure to the biocidal agent), and the percentage reduction (%) after exposure to the biocidal agent in the disinfection chamber for 10 and 30 s for each test microorganism and for each individual PPE item evaluated in this study. Fig 3 shows the graphs of the logarithmic reduction (log_10_) of each test microorganism per individual PPE item.

**Table 2:** Result of the number of viable cells in CFU/mL and their equivalent number in (CFU/cm^2^), log_10_ reduction factor (mean ± standard deviation) and percentage reduction for the microorganisms studied at 10 and 30 s of exposure to the biocidal agent in the disinfection chamber (results obtained from the comparative analysis with the control without exposure to the biocidal agent).

Test microorganisms	Exposure conditions	Personal Protection Equipment													
		Cap		Safety glasses		Respirator face mask		Lab coat		Glove		Shoe		Face shield	
		Log_10_ and (% reduction)	Number of viable cells in CFU/mL and (CFU/cm^2^)	Log_10_ and (% reduction)	Number of viable cells in CFU/mL and (CFU/cm^2^)	Log_10_ and (% reduction)	Number of viable cells in CFU/mL and (CFU/cm^2^)	Log_10_ and (% reduction)	Number of viable cells in CFU/mL and (CFU/cm^2^)	Log_10_ and (% reduction)	Number of viable cells in CFU/mL and (CFU/cm^2^)	Log_10_ and (% reduction)	Number of viable cells in CFU/mL and (CFU/cm^2^)	Log_10_ and (% reduction)	Number of viable cells in CFU/mL and (CFU/cm^2^)
*E*. *faecalis*	Control	7.78±0.05	6.0x10^7^	7.77±0.02	5.9x10^7^	8.72±0.01	5.3x10^8^	8.74±0.02	5.5x10^8^	4.30±0.03	2.0x10^4^	8.76±0.01	5.7x10^8^	7.61±0.03	4.1x10^7^
			(2.0x10^6^)		(2.0x10^6^)		(1.8x10^7^)		(1.8x10^7^)		(6.7x10^2^)		(1.9x10^7^)		(1.4x10^6^)
	10 s	3.36^g^±0.08 (>99%)	2.6x10^4^	3.45^g^±0.03 (>99%)	2.1x10^4^	1.96^g^±0.02 (98.906%)	5.8x10^6^	4.96^f^±0.06 (>99%)	6.0x10^3^	0.87^g^±0.10 (86.500%)	2.7x10^3^	4.79^f^±0.01 (>99%)	9.2x10^3^	4.06^f^±0.05 (>99%)	3.6x10^3^
			(8.7x10^2^)		(7.0x10^2^)		(1.9x10^6^)		(2.0x10^2^)		(9.0x10^1^)		(3.1x10^2^)		(1.2x10^2^)
	30 s	4.00^f^±0.05 (>99%)	6.0x10^3^	3.79^f^±0.02 (>99%)	9.6x10^3^	2.09^f^±0.03 (>99%)	4.3x10^6^	5.42^e^±0.06 (>99%)	2.1x10^3^	0.85^g^±0.0 (86.000%)	2.8x10^3^	5.08^e^±0.03 (>99%)	4.7x10^3^	4.15^e^±0.09 (>99%)	2.9x10^3^
			(2.0x10^2^)		(3,2x10^2^)		(1.4x10^5^)		(7.0x10^1^)		(9.3x10^1^)		(1.6x10^2^)		(9.7x10^1^)
*P*. *aeruginosa*	Control	8.66±0.04	4.6x10^8^	8.66±0.03	4.6x10^8^	8.72±0.02	5.3x10^8^	8.84±0.01	6.9x10^8^	8.74±0.03	5.5x10^8^	8.77±0.02	5.9x10^8^	8.82±0.05	6.6x10^8^
			(1.5x10^7^)		(1,5x10^7^)		(1.8x10^7^)		(2.3x10^7^)		(1.8x10^7^)		(2.0x10^7^)		(2.2x10^7^)
	10 s	2.78^i^±0.04 (>99%)	7.7x10^5^	2.81^h^±0.04 (>99%)	7.2x10^5^	2.92^e^±0.04 (>99%)	6.4x10^5^	8.84^b^±0.01 (>99%)	<10	8.74^b^±0.03	<10	2.89^h^±0.04 (>99%)	7.6x10^5^	8.82^b^±0.05 (>99%)	<10
			(2,6x10^4^)		(2,4x10^4^)		(2.1x10^4^)		(<0.33)	(>99%)	(<0.33)		(2.5x10^4^)		(<0.33)
	30 s	2.80^h^±0.05 (>99%)	7.3x10^5^	2.76^i^±0.03 (>99%)	8.0x10^5^	2.93^e^±0.05 (>99%)	6.2x10^5^	8.84^b^±0.01 (>99%)	<10	8.74^b^±0.03 (>99%)	<10	8.77^b^±0.02 (>99%)	<10	8.82^b^±0.05 (>99%)	<10
			(2.4x10^4^)		(2,7x10^4^)		(2.1x10^4^)		(<0.33)		(<0.33)		(<0.33)		(<0.33)
*C*. *freundii and P*. *mirabilis*	Control	8.26±0.03	1.8x10^8^	8.26±0.09	1.8x10^8^	8.28±0.06	1.9x10^8^	8.26±0.02	1.8x10^8^	8.26±0.01	1.8x10^8^	8.28±0.03	1.9x10^8^	8.23±0.07	1.7x10^8^
			(6.0x10^6^)		(6,0x10^6^)		(6.3x10^6^)		(6.0x10^6^)		(6.0x10^6^)		(6.3x10^6^)		(5,7x10^6^)
	10 s	8.26^b^±0.03 (>99%)	<10	8.26^b^±0.09 (>99%)	<10	8.28^b^±0.06 (>99%)	<10	8.26^c^±0.02 (>99%)	<10	8.26^c^±0.01 (>99%)	<10	8.28^c^±0.03 (>99%)	<10	8.23^c^±0.07 (>99%)	<10
			(<0.33)		(<0.33)		(<0.33)		(<0.33)		(<0.33)		(<0.33)		(<0.33)
	30 s	8.26^b^±0.03 (>99%)	<10	8.26^b^±0.09 (>99%)	<10	8.28^b^±0.06 (>99%)	<10	8.26^c^±0.02 (>99%)	<10	8.26^c^±0.01 (>99%)	<10	8.28^c^±0.03 (>99%)	<10	8.23^c^±0.07 (>99%)	<10
			(<0.33)		(<0.33)		(<0.33)		(<0.33)		(<0.33)		(<0.33)		(<0.33)
*E*. *coli*	Control	9.56±0.02	3.6x10^9^	9.58±0.02	3.8x10^9^	9.61±0.04	4.1x10^9^	9.69±0.04	4.9x10^9^	9.72±0.02	5.3x10^9^	9.59±0.05	3.9x10^9^	9.65±0.05	4.5x10^9^
			(1.2x10^8^)		(1,3x10^8^)		(1.4x10^8^)		(1.6x10^8^)		(1.8x10^8^)		(1.3x10^8^)		(1.5x10^8^)
	10 s	9.56^a^±0.02 (>99%)	<10	9.58^a^±0.02 (>99%)	<10	9.61^a^±0.04 (>99%)	<10	9.69^a^±0.04 (>99%)	<10	9.72^a^±0.02 (>99%)	<10	9.59^a^±0.05 (>99%)	<10	9.65^a^±0.05 (>99%)	<10
			(<0.33)		(<0.33)		(<0.33)		(<0.33)		(<0.33)		(<0.33)		(<0.33)
	30 s	9.56^a^±0.02 (>99%)	<10	9.58^a^±0.02 (>99%)	<10	9.61^a^±0.04 (>99%)	<10	9.69^a^±0.04 (>99%)	<10	9.72^a^±0.02 (>99%)	<10	9.59^a^±0.05 (>99%)	<10	9.65^a^±0.05 (>99%)	<10
			(<0.33)		(<0.33)		(<0.33)		(<0.33)		(<0.33)		(<0.33)		(<0.33)
*S*. *aureus*	Control	7.57±0.01	3.7x10^7^	7.63±0.03	4.3x10^7^	7.54±0.03	3.5x10^7^	7.60±0.02	4.0x10^7^	7.43±0.05	2.7x10^7^	7.59±0.05	3.9x10^7^	7.32±0.06	2.1x10^7^
			(1.2x10^6^)		(1.3x10^6^)		(1.2x10^6^)		(1.3x10^6^)		(9.0x10^5^)		(1.3x10^6^)		(7x10^5^)
	10 s	7.57^c^±0.01 (>99%)	<10	7.63^c^±0.03 (>99%)	<10	7.54^c^±0.03 (>99%)	<10	7.60^d^±0.02 (>99%)	<10	7.43^d^±0.05 (>99%)	<10	7.59^d^±0.05 (>99%)	<10	7.32^d^±0.06 (>99%)	<10
			(<0.33)		(<0.33)		(<0.33)		(<0.33)		(<0.33)		(<0.33)		(<0.33)
	30 s	7.57^c^±0.01 (>99%)	<10	7.63^c^±0.03 (>99%)	<10	7.54^c^±0.03 (>99%)	<10	7.60^d^±0.02 (>99%)	<10	7.43^d^±0.05 (>99%)	<10	7.59^d^±0.05 (>99%)	<10	7.32^d^±0.06 (>99%)	<10
			(<0.33)		(<0.33)		(<0.33)		(<0.33)		(<0.33)		(<0.33)		(<0.33)
*C*. *albicans*	Control	4.40±0.05	2.5x10^4^	5.00±0.06	1.0x10^5^	4.00±0.06	1.0x10^4^	4.60±0.03	4.0x10^4^	4.54±0.05	3.5x10^4^	4.30±0.03	2.0x10^4^	4.08±0.05	1.2x10^4^
			(8.3x10^2^)		(3.3x10^3^)		(3.3x10^2^)		(1.3x10^6^)		(1.2x10^3^)		(6.8x10^2^)		(4.0x10^2^)
	10 s	4.40^d^±0.05 (>99%)	<10	5.00^e^±0.06 (>99%)	<10	4.00^d^±0.06 (>99%)	<10	4.60^g^±0.03 (>99%)	<10	4.54^e^±0.05 (>99%)	<10	4.30^g^±0.03 (>99%)	<10	4.08^f^±0.05 (>99%)	<10
			(<0.33)		(<0.33)		(<0.33)		(<0.33)		(<0.33)		(<0.33)		(<0.33)
	30 s	4.40^d^±0.05 (>99%)	<10	5.00^e^±0.06 (>99%)	<10	4.00^d^±0.04 (>99%)	<10	4.60^g^±0.03 (>99%)	<10	4.54^e^±0.05 (>99%)	<10	4.30^g^±0.03 (>99%)	<10	4.08^f^±0.05 (>99%)	<10
			(<0.33)		(<0.33)		(<0.33)		(<0.33)		(<0.33)		(<0.33)		(<0.33)
*C*. *parapsilosis*	Control	4.18±0.04	1.5x10^4^	5.48±0.04	3.0x10^5^	4.00±0.04	1.0x10^4^	4.30±0.03	2.0x10^4^	4.20±0.08	1.6x10^4^	4.30±0.06	2.0x10^4^	4.18±0.07	1.5x10^4^
			(5x10^2^)		(1.0x10^4^)		(3.3x10^2^)		(6.8x10^2^)		(5.3x10^2^)		(6.8x10^2^)		(5.0x10^2^)
	10 s	4.18^e^±0.04 (>99%)	<10	5.48^d^±0.04 (>99%)	<10	4.00^d^±0.04 (>99%)	<10	4.30^h^±0.03 (>99%)	<10	4.20^f^±0.08 (>99%)	<10	4.30^g^±0.06 (>99%)	<10	4.18^e^±0.07 (>99%)	<10
			(<0.33)		(<0.33)		(<0.33)		(<0.33)		(<0.33)		(<0.33)		(<0.33)
	30 s	4.18^e^±0.04 (>99%)	<10	5.48^d^±0.04 (>99%)	<10	4.00^d^±0.04 (>99%)	<10	4.30^h^±0.03 (>99%)	<10	4.20^f^±0.08 (>99%)	<10	4.30^g^±0.06 (>99%)	<10	4.18^e^±0.07 (>99%)	<10
			(<0.33)		(<0.33)		(<0.33)		(<0.33)		(<0.33)		(<0.33)		(<0.33)

In total, 147 experimental conditions were studied, considering the two exposure times for each test microorganism (n = 98) and control (n = 49), as well as for the seven different types of PPE evaluated. In general, there was a significant reduction in the investigated microorganisms after exposure of the previously contaminated items in the disinfection chamber, regardless of the item, which demonstrated the efficacy of the biocidal agent spraying system for instantaneous disinfection of PPE for some of the microorganisms evaluated. In addition, the results showed that at 10 and 30 s, there was only a significant difference in the microbial load reduction factor during exposure of the biocidal agent to the microorganisms *E*. *faecalis* and *P*. *aeruginosa* (p >0.05). In this case, the exposure time of 30 s was more efficient for the inactivation of the studied microorganisms in terms of log_10_ and percentage reduction for all studied PPE (Table 2 and Fig 3).

A percentage reduction of >99% was determined for 96.93% (n = 95) of the tested conditions when compared to the control, while a percentage reduction of between 86.00–99% was found for 3.07% (n = 3) of the tested conditions. The lowest percentage reductions identified were 86.500 and 86.000% for the test with *E*. *faecalis* when the glove was evaluated at exposure times of 10 and 30 s, respectively. In general, the exposure time of 10 s to 0.25% sodium hypochlorite under the investigated conditions effectively reduced the microbial load by >99% for all investigated microorganisms, except for *E*. *faecalis*. For these microorganism, percentage reduction >99% at 10 s and 30 s of exposure was identified for all PPE, except for the glove and respirator face mask. It is noteworthy that the results related to the percentage reduction for the exposure time of 30 s were similar to the time of 10 s, except for *E*. *faecalis* (Table 2).

The percentage reduction is also reflected in the total number of viable cells, where 78.57% (n = 77) of the analyzed conditions corresponded to <10 CFU/mL or <0.33 CFU/cm^2^ at 10 and 30 s of exposure to spraying of 0.25% sodium hypochlorite. In general, there was a reduction in the number of viable cells for all analyzed conditions when compared to the results found for the control group. The resistance to the biocidal agent under the investigated conditions of the microorganisms *E*. *faecalis* and *P*. *aeruginosa* was demonstrated in this parameter, since they were the only ones that showed viable cells in the concentration >10 CFU/mL or >0.33 CFU/cm^2^ after the disinfection process in the chamber, with the exception of samples of *P*. *aeruginosa* collected from the face shield, glove and lab coat, at exposure times of 10 and 30 s, in addition to the shoe after 30 s of exposure. The exposure time of 10 s was able to reduce the number of viable cells for <10 CFU/mL or <0.33 CFU/cm^2^ in 38 experimental conditions, while for the time of 30 s, this reduction occurred in 39 experimental conditions. The differences related to the recovery of microorganisms may be associated with resistance to the biocidal agent under the conditions tested, as well as the inoculum concentration.

Fig 3 shows that *E*. *coli* was the microorganism with the highest log_10_ reduction values, regardless of the analyzed experimental condition (PPE item/surface and exposure time), with values >9 log_10_. The microorganisms *S*. *aureus*, *C*. *albicans*, *C*. *parapsilosis*, *C*. *freundii* and *P*. *mirabilis* showed the same log_10_ reduction at the tested exposure times, without significant difference regardless of the surface analyzed. It is important to highlight that for *Candida* species the log_10_ reduction value was lower than the bacterial species and this effect may be due to the lower initial inoculum used. With regard to the PPE items, the lab coat showed the highest log_10_ reduction values, varying between 4.30 log_10_ (*C*. *parapsilosis* at 10 and 30 s) and 9.69 log_10_ (*E*. *coli* at 10 and 30 s). The surface of the glove was the only one in which the log_10_ reduction of *E*. *faecalis* was similar (p >0.05) at the exposure times of 10 and 30 s, with 0.87 and 0.85 log_10_, respectively, and was the lowest when compared to the reduction in the other analyzed surface.

**Fig 4:**
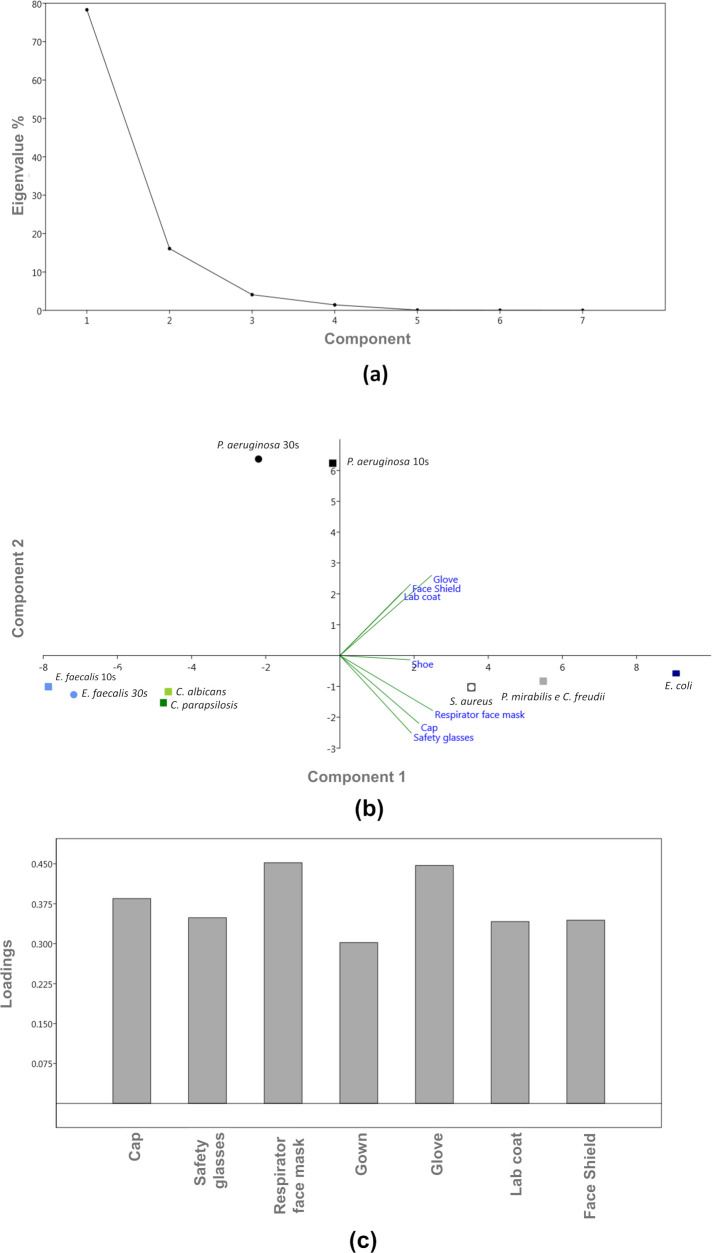
Principal component analysis of the samples of the test microorganisms analyzed at 10 and 30 s: (a) cumulative variance according to the quantity of components (PPE–cap, safety glasses, respirator face mask, lab coat, glove, shoe and face shield—or surface type) (%); (b) graph of scores of principal components 1 (respirator face mask) and 2 (glove) and (c) graph of variable loadings of principal component 1.

Fig 4 shows the results of the PCA applied to the different study variables, which in this study were related to the type of PPE (cap, safety glasses, respirator face mask, lab coat, glove, shoe and face shield). In PCA, the clustering of the samples defines the structure of the data through graphs of scores and loadings whose axes are principal components (PC) on which the data is projected. The scores provide the composition of the PCs in relation to the samples, while loadings provide that same composition in relation to the variables. The total variance of principal component 1, mainly influenced by the respirator face mask, was 78.31% and that of principal component 2, mainly influenced by the glove, was 16.01%, totaling 94.32% (Fig 4A). The graph of the principal components shows that the microorganisms *E*. *faecalis* and *P*. *aeruginosa* were the only ones for which the samples from the 10 and 30 s exposure times did not overlap (Fig 4B). In addition, the graph shows the formation of clusters for all tested microorganisms at different exposure times. Clustering is important because it can indicate similar behavior among the samples evaluated according to the study variables. Note that the only analyzed fungi (*C*. *albicans* and *C*. *parapsilosis*) formed a cluster in the lower left quadrant, as well as the samples from 10 and 30 s of *E*. *faecalis*, having no influence on the type of surface/item tested. However, the microorganisms *S*. *aureus*, *E*. *coli*, *C*. *freundii* and *P*. *mirabilis* clustered together in the lower right quadrant, being influenced by the type of surface/item tested (safety glasses, cap, respirator face mask and shoe), indicating that these microorganisms had similar behavior at the evaluated experimental conditions.

*P*. *aeruginosa* was the only microorganism whose samples from 10 and 30 s were allocated in the upper right quadrant, not being influenced by the main analyzed variable, the PPE item. Note also that there was no negative correlation between the porous and nonporous surface variables for principal component 1, where all PPE correlated positively with each other (Fig 4c). Thus, from the PCA analysis, it is observed that the type of microorganism analyzed influenced the results, since there was formation of clusters. In addition, even for the microorganisms that did not have overlapping values for the exposure times of 10 and 30 s, the behavior in response to the variables was similar since they remained in the same quadrant.

**Fig 5:**
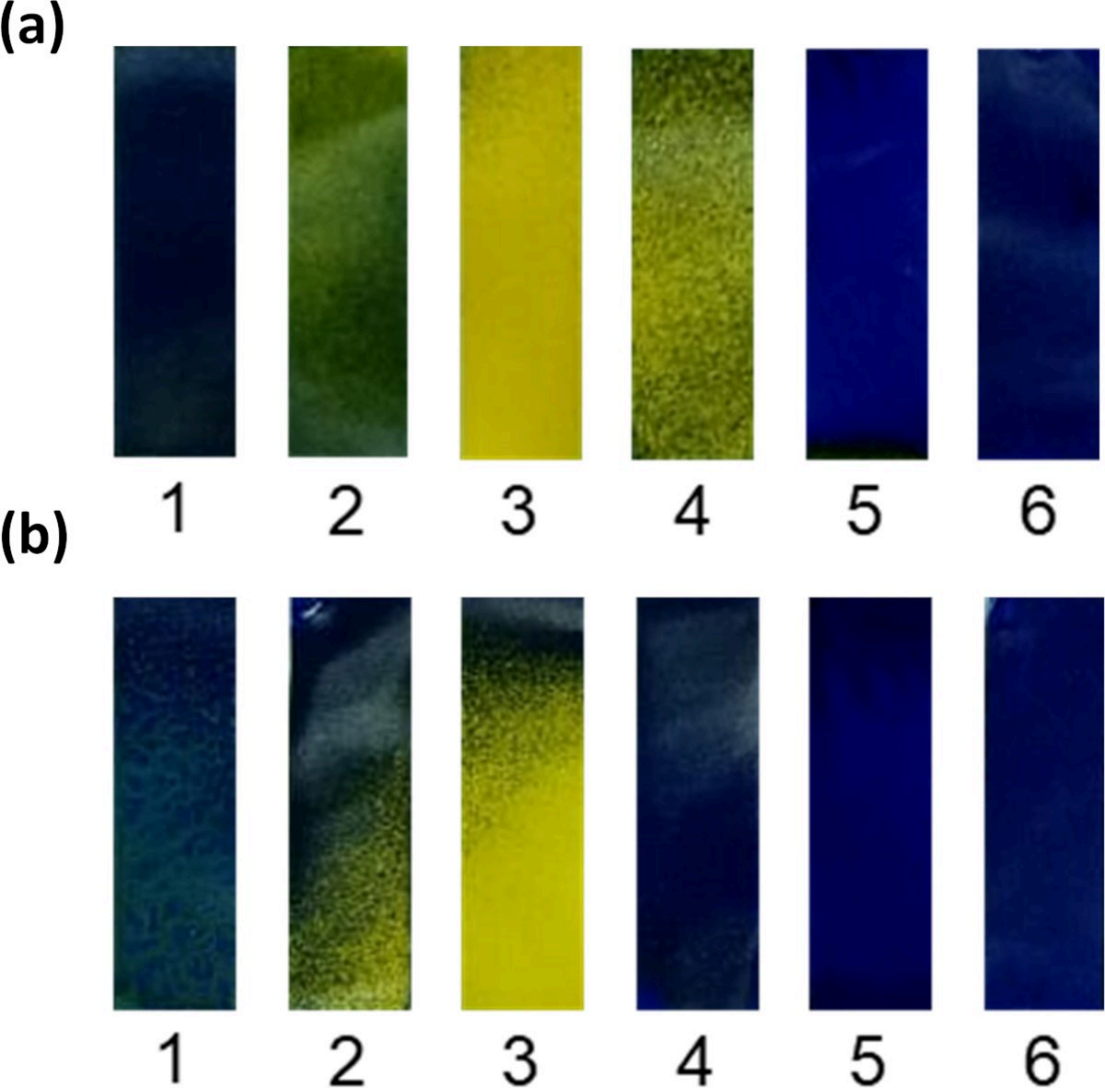
Discoloration of the WSPs exposed to spraying of the biocidal agent in the disinfection chamber in the study areas: (a) WSPs collected after 10 s of exposure; (b) WSPs collected after 30 s of exposure. WSPs position: (1) cap; (2) safety glasses; (3) respirator face mask; (4) lab coat; (5) glove; and (6) shoe. Blue areas represent the deposition of the biocidal agent, while yellow areas indicate no deposition.

Fig 5 shows images of WSP discoloration due to the absorption of sodium hypochlorite droplets sprayed in the disinfection chamber after 10 and 30 s of exposure. Each image was observed ex situ after the WSPs were removed from the study surface areas (positions 1 to 6—Fig 2). In general, there was good dispersion of the biocidal agent across the study area when using the disinfection chamber composed of the six nebulizer nozzles. The areas of the WSP with bluish tones show that there was deposition of the biocidal agent during the passage of the manikin through the disinfection chamber, while areas with yellowish tones indicate the absence of deposition of the studied agent.

In general, when comparing the distribution of the biocidal agent at the investigated exposure times, similar good deposition coverage of the agent on the WSPs was observed, which may be associated with the log_10_ reduction profile of the studied microorganisms, where the exposure time to the biocidal agent had no significant influence on the reduction factor (except for *E*. *faecalis* and *P*. *aeruginosa*) (Table 2 and Fig 3). Thus, the amount of biocidal agent that reaches the study areas during the 10 s exposure would be sufficient to inactivate most of the investigated microorganisms. Qualitatively, greater deposition is observed in some points of the WSPs for the time of 30 s and this may explain the greater efficacy of the longer exposure time for the two most resistant microorganisms (*E*. *faecalis* and *P*. *aeruginosa*). In addition, the lower deposition of the biocidal agent on the respirator face mask (area 3), indicated by the yellowish tone, may have interfered with the reduction efficiency of these microorganisms.

**Fig 6:**
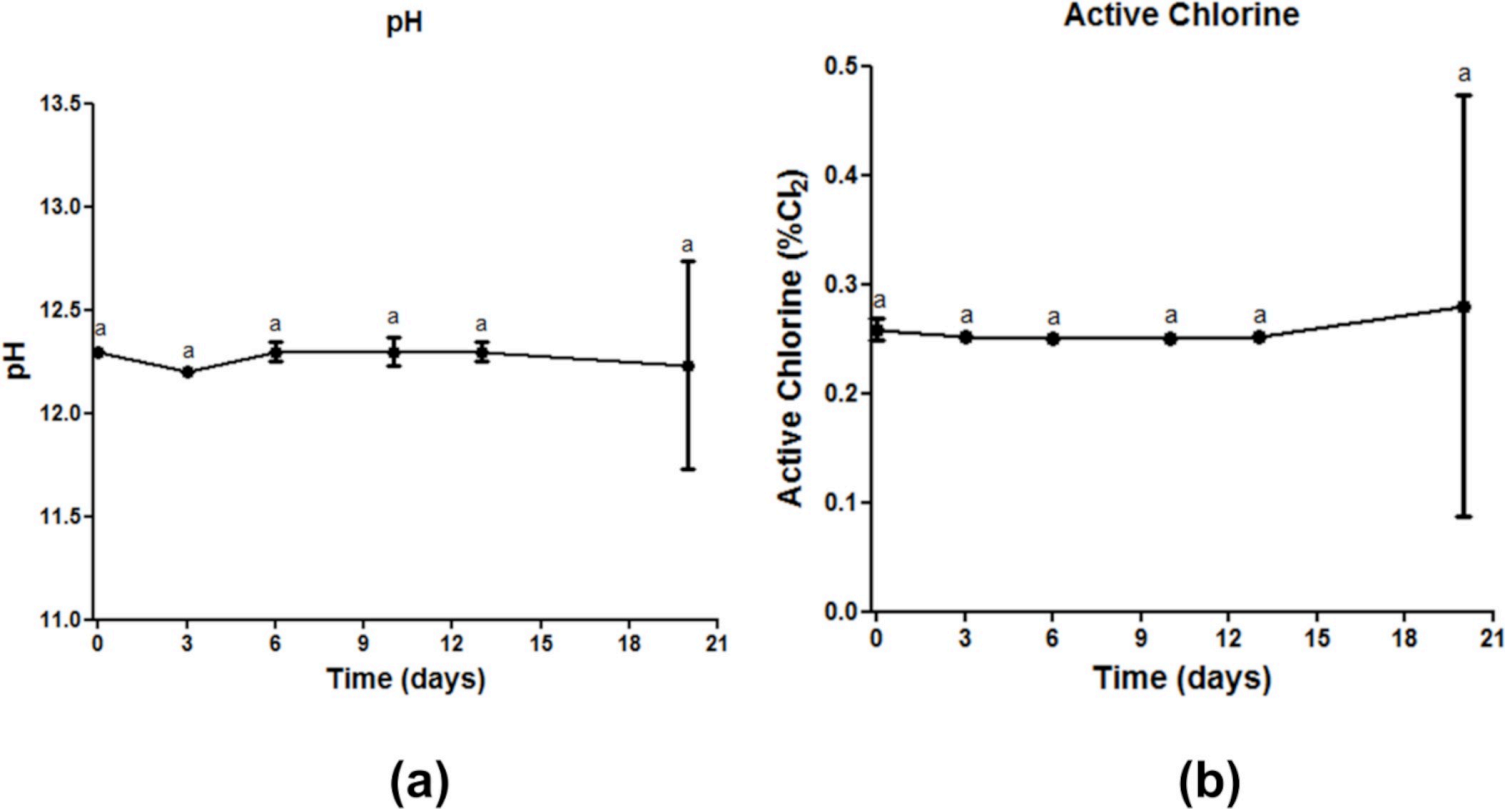
Stability analysis of sodium hypochlorite on days 0, 3, 6, 10, 13 and 20: (a) pH analysis and (b) percentage of active chlorine. There was no significant difference at p <0.05 between values followed by the same letter according to Student’s t test with 95% confidence.

Fig 6 and S1 Table shows the results obtained for the stability analysis of the biocidal agent at a concentration of 0.25% by determining the pH (Fig 6A) and the percentage of active chlorine (Fig 6B). The results showed that the sodium hypochlorite solution was stable over the evaluated period, with no significant difference (p >0.05) between the means determined for the pH and for the active chlorine concentration (%). These results demonstrate the viability of using the sodium hypochlorite solution with adequate active chlorine concentration (0.25%) for at least 20 days for application in the disinfection chamber, with maintenance of its disinfectant capacity.

## Discussion

In this study we demonstrated the high rates of microbial load reduction after exposure to the sodium hypochlorite biocidal agent used in the personal protective closes and equipment (PPE) disinfection chamber at the two analyzed times (10 and 30 s) regardless of the type of surface/PPE item investigated. Some studies [80, 81] and standards [79, 82] indicate that disinfection methods with ≥5 log_10_ CFU reduction are considered effective and, consequently, appropriate for clinical use, which reinforces the importance of our results for the instant disinfection of PPE, especially during the SARS-CoV-2 pandemic. This logarithmic reduction implies the elimination of 99.999% of the microbial load [79, 82]. Considering these values as a reference, the microorganisms *P*. *aeruginosa* and *E*. *faecalis* showed the lowest sensitivity to sodium hypochlorite under the tested conditions (for some PPE items) when compared to the other microorganisms, although the results were quite satisfactory in relation to the reduction factor found for these bacteria under the studied conditions.

There are reports in the literature on the resistance of *P*. *aeruginosa* and *E*. *faecalis* to sodium hypochlorite at concentrations <0.3% and <0.22%, respectively [83, 84]. This mechanism may be associated with the bacterial ability to remove or discharge the charge of hypochlorous acid (HClO), which is a strong oxidizing agent that damages the permeability of the bacterial cell wall and its genetic material [85]. However, Lineback et al. [86] showed that the use of sodium hypochlorite at a concentration of 1.312% against *P*. *aeruginosa* was more effective than quaternary ammonium, while Yoo et al. [87] reported that this biocidal agent at 0.031% showed activity against clinical isolates of *E*. *faecalis*. In this study, the concentration of 0.25% of sodium hypochlorite was effective in reducing the load >99% for *P*. *aeruginosa* and ≥86.000% for *E*. *faecalis*.

Regarding the analyzed fungal strains, the percentage reduction value was >99% and the number of viable cell was <10 CFU/mL or <0.33 CFU/cm^2^ for all experimental conditions, indicating that *C*. *albicans* and *C*. *parapsilosis* are sensitive to sodium hypochlorite under the tested conditions, which shows that spraying of the biocidal agent may be an effective alternative for the inactivation of these microorganisms when compared to other methods [62]. Infections caused by *Candida* species are classified as one of the main contaminants in the hospital environment because these pathogens can lead to systemic infection [88]. Although *C*. *albicans* is still the species most frequently isolated from nosocomial fungal infections [89], cases associated with *C*. *parapsilosis* have increased significantly in recent years [90] due to the resistance of *Candida* species to antifungals and disinfectants [91]. Thus, it is important to note that spraying systems have been used to control bioburden in nosocomial environments, especially for combating multidrug-resistant strains [81].

The study by Ishikawa et al. [37] demonstrated that the efficacy of a small disinfection chamber using a spray system with 5.00% sodium hypochlorite solution for the inactivation of *Bacillus subtilis* spores. The authors reported that the disinfection system used is a "test chamber", which does not have the physical structure for the passage of a person, being able only to perform the sporicidal effect in a small area [37]. However, unlike Ishikawa et al. [37] work, our study demonstrates the efficacy of a spray system (disinfection chamber) containing sodium hypochlorite for the instant disinfection of different PPE items (at 10 or 30 s) against *Candida* species and Gram-positive and Gram-negative bacteria on different types of surfaces at the same time. The efficacy demonstrated by the biocide agent in the concentration of 0.25% against the microorganisms tested suggests that new studies can be conducted using a lower concentration of the chemical agent, such as 0.1%, which is also in the concentration range recommended by WHO for the disinfection of environmental surfaces [49].

Hospital infections are caused by factors such as environmental contamination, frequent handling of contaminated material, and the ability of microorganisms to survive for prolonged periods on different types of surfaces [1, 92]. Within the hospital environment, sodium hypochlorite is the most widely used disinfectant because it has broad-spectrum antimicrobial activity, considering Gram-positive and Gram-negative bacteria and fungi [83] as well as demonstrated virucidal activity [93]. Köhler et al. [94] showed that sodium hypochlorite effectively reduced the concentration of multidrug-resistant Gram-positive bacteria (*Pseudomonas*, *Acinetobacter* and *Klebsiella*) after exposure times of 1 to 15 min, longer exposure time than those studied in this work. Regarding the activity against viral agents, It has been demonstrated that sodium hypochlorite in concentrations between 0.01 and 0.5% is capable of inactivating the SARS-CoV-1 on stainless steel surfaces [95, 96]. In addition, Ma et al. [97] showed that instant hand hygiene using disinfecting wipes containing 0.05 or 0.25% active chlorine removed 96.62% and 99.98%, of the influenza virus, respectively, which causes avian influenza. The authors also point out that, although they have not tested with SARS-CoV-2, hand cleaning with the disinfecting wipes can help to control the spread of COVID-19. Similarly to the study by Ma et al. [97], one limitation of our study was not to use SARS-CoV-2 as a test microorganism. However, based on the promising results identified in this study, which showed that the biocide capacity of sodium hypochlorite was maintained under the conditions tested, the evaluated concentration of 0.25% has the potential for disinfecting surfaces contaminated by different microorganisms and can be extrapolated to enveloped viruses based on results in the literature, being a potential agent against SARS-CoV-2. The choice of evaluating different bacteria and fungi as biological indicators stemmed from the need to accelerate the confirmation of the disinfectant action of the proposed technology, since some of these microorganisms are resilient compared to viruses [98], have a relatively faster growth and can be manipulated in laboratory environments with a lower level of biosafety.

Other studies have already demonstrated the effect of different biocidal agents against the microorganisms tested in this study, showing that the efficacy of the disinfection process varies with the type of disinfectant [99, 100] or according to the application method used [101, 102]. There are few reports in the scientific literature on the efficacy of PPE decontamination after exposure to pathogens, as was analyzed in this study. Among them, Lemmer et al. [103] showed that disinfection with 2% peracetic acid with 0.2% surfactant through a spray system was able to inactivate *B*. *thuringiensis* spores in high density polyethylene protective coveralls after 5 min of exposure. Compared to our study, sodium hypochlorite was more effective than peracetic acid because the exposure time required was shorter to reduce the microbial load on the surface, considering the polyethylene surface and other analyzed materials. In addition, the instantaneous decontamination demonstrated by the results obtained in 10 and 30 s exposure times shows the potential of the disinfection chamber application in places with intense or moderate people flow, such as the exits of intensive care units and wards in hospitals. In addition, reducing the microbial load on the surface of PPE can help reduce the risks associated with handling and exposure to biomedical waste, an important source of environmental contamination [104].

The results also suggest that the use of sodium hypochlorite may be recommended due to the stability of its solution in terms of pH and concentration of active chlorine over the 20 days, since the exchange or replacement of the biocide agent solution does not need to be performed, for example, on a daily basis. The dissociation of NaOCl into HOCl-, its main active agent, is pH-dependent [105]. Thus, it is important that the solution remains stable so that the levels of the active agent do not decrease. The critical issue raised by health authorities’ agencies are sodium hypochlorite toxicity when in contact with mucous membranes and may lead to tissue damage or allergic reactions [106, 107]. Therefore, we are aware of the possibility of episodes of clinical toxicity caused by the sodium hypochlorite, especially in persons that are known to be allergic to bleaches, and that this can be considered as a limitation of the study. In addition, we re-emphasized that the disinfection chamber with 0.25% sodium hypochlorite be used only by fully trained workers (considering its installation, the preparation of the biocide agent in the correct concentration, and for its correct use), thus promoting an effective disinfection process against bacteria and potential emerging pathogens, such as SARS-CoV-2, that is safe for users.

The use of the proposed disinfection technology becomes an attractive alternative, especially for middle-income countries such as Brazil [38]. This occurs due to factors such as the material used in the chamber framework, which are widely used in the industry for the manufacture of different items, its modular design that allows scalable production as well as the low-cost of sodium hypochlorite. These factors can make production cheaper and facilitate transport and installation in different health facilities. In addition, the results obtained related to WPS showed that the nebulizer nozzles disposition promoted a satisfactory spraying of the biocide agent.

Indeed, disinfection chamber could be considered as an interesting disinfection technology for use in emerging countries, since it can improve the control cases of nosocomial infection in those places that usually have the most overloaded health systems.

## Conclusions

The disinfection chamber proved to be a potential technology for the rapid and effective disinfection of the surface of PPE, regardless of the evaluated item, routinely used by HCWs for protection against infectious agents. The spraying system with the biocidal agent was effective in reduce the microbial load, where the percentage reduction equal to >99% and, consequently, bringing the number of viable cells to <10 CFU/mL and <0.33 CFU/cm^2^ after exposure times of 10 and 30 s in 96.93% of the experimental conditions analyzed. The lowest percentages reduction were found for the sample of *E*. *faecalis* collected from the glove, where the values obtained were 86.000 and 86.500% for the exposition of 10 and 30 s, respectively, while the highest amount of viable cells was found for *P*. *aeruginosa* sample at 10 s in the cap, with 7.7x10^5^ CFU/mL or 2.6x10^4^ CFU/cm^2^. The log_10_ reduction values varied between 0.85 log_10_ (*E*. *faecalis* at 30 s in glove surface) and 9.69 log_10_ (*E*. *coli* at 10 and 30 s in lab coat surface).

Thus, the bacterial species *E*. *coli*, *S*. *aureus*, *C*. *freundii* and *P*. *mirabilis* and the fungi *C*. *albicans* and *C*. *parapsilosis* showed susceptibility to 0.25% sodium hypochlorite under the evaluated experimental conditions independent of the exposure time or PPE item evaluated, while the microorganism *E*. *faecalis* was less susceptible to the biocidal agent under the tested conditions. In general, a 30-s exposure time was more efficient in reducing the investigated microbial load.

The results of this study show that the disinfection chamber with 0.25% sodium hypochlorite may be an alternative to control the bioburden in nosocomial environments, especially to prevent the self-contamination of HCWs in the doffing step. The importance of the use of the chamber by properly attired HCWs is also emphasized in order to avoid direct contact with the tested biocidal agent. In addition, because this is a novel study, these results may contribute to the development and safe use of disinfection equipment in environments where the environmental bioburden must be controlled. It is also important to highlight that the experimental design of the study was carried out in order to have a simulation of how the disinfection chamber would be used by HCW in the nosocomial environment. Thus, the manipulation of viral strains would not be appropriate in the conditions tested, since the handling of these microorganisms requires a laboratory environment with a higher level of biosafety. However, although virucidal efficacy was not directly determined, the chamber may be an alternative to reduce the contamination rates among HCWs in front of different types of emerging microorganisms, reducing the impacts in the area of public health.

## Supporting information

S1 Fig Image of the disinfection chamber: Spray disinfection technology for instant decontamination of personal protective equipment. (DOCX)

S2 Fig Images of the manikin suitably dressed with PPEs used to simulate the use of the disinfection chamber by healthcare workers in nosocomial environments. (DOCX)

S1 Table Raw data of stability of sodium hypochlorite regarding pH and active chlorine analysis (mean ± standard deviation). (DOCX)
