# Supplementary material for: Potential application of novel technology developed for instant decontamination of personal protective equipment before the doffing step
Source: PLoS One. 2021 Jun 4;16(6):e0250854. doi: 10.1371/journal.pone.0250854 (PMC8177472; doi:10.1371/journal.pone.0250854)
Supplement: S1 Fig — (DOCX) [file pone.0250854.s001.docx]

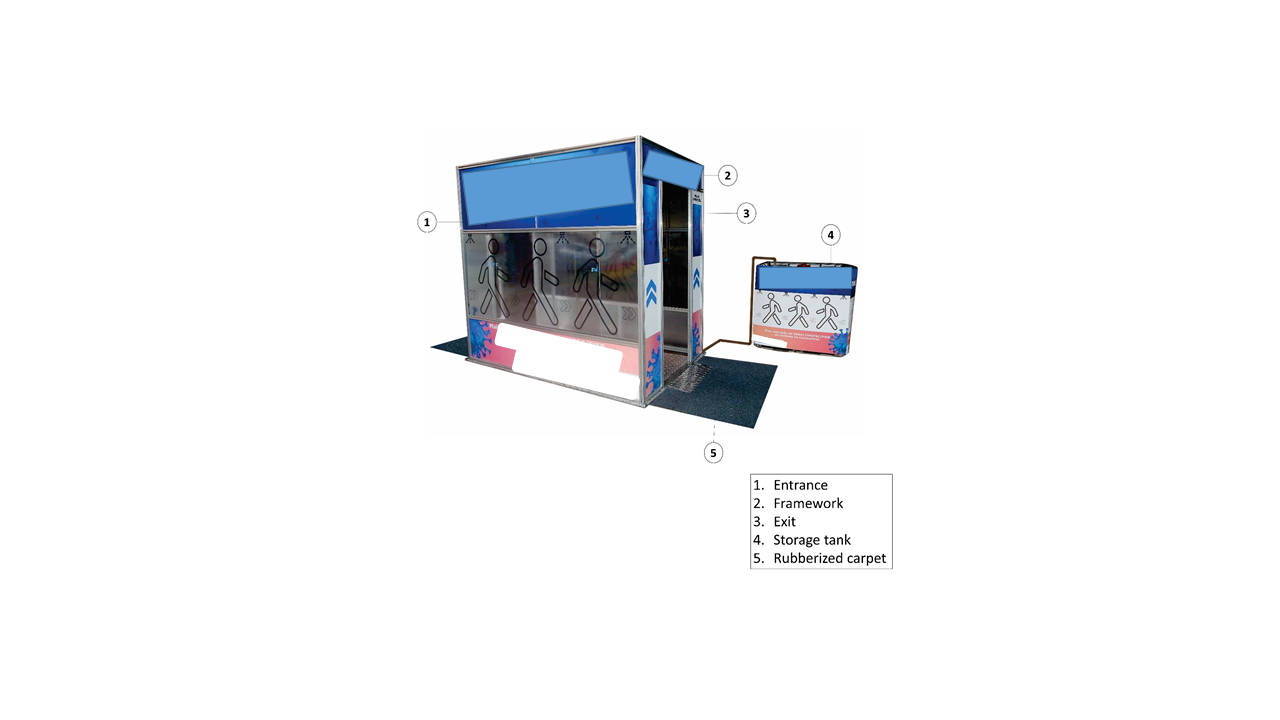


**S1 Fig**. Image of the disinfection chamber: spray disinfection technology for instant decontamination of personal protective equipment.
