# Supplementary material for: Potential application of novel technology developed for instant decontamination of personal protective equipment before the doffing step
Source: PLoS One. 2021 Jun 4;16(6):e0250854. doi: 10.1371/journal.pone.0250854 (PMC8177472; doi:10.1371/journal.pone.0250854)
Supplement: S1 Table — (DOCX) [file pone.0250854.s003.docx]

**S1 Table**. Raw data of stability of sodium hypochlorite regarding pH and active chlorine analysis (mean ± standard deviation).

| **Time (days)** | **pH** | **Active chlorine (%)** |
| --- | --- | --- |
| 0 | 12.3 ± 0.02 | 0.259 ± 0.01 |
| 3 | 12.2 ± 0.01 | 0.252 ± 0.001 |
| 6 | 12.3 ± 0.05 | 0.251 ± 0.001 |
| 10 | 12.3 ± 0.07 | 0.251 ± 0.001 |
| 13 | 12.3 ± 0.05 | 0.252 ± 0.005 |
